# Halophilic Pectinase-Producing Bacteria from *Arthrocnemum macrostachyum* Rhizosphere: Potential for Fruit–Vegetable Juice Processing

**DOI:** 10.3390/microorganisms12112162

**Published:** 2024-10-26

**Authors:** Amal S. Alswat, Ohud Muslat Alharthy, Seham Saeed Alzahrani, Seham Sater Alhelaify

**Affiliations:** Department of Biotechnology, College of Science, Taif University, P.O. Box 11099, Taif 21944, Saudi Arabia

**Keywords:** antioxidant activities, detox juices, salt tolerance, total phenolics

## Abstract

This study aimed to isolate salt-tolerant pectinolytic bacteria from the rhizosphere of a salt marsh plant and utilize their pectinases for the clarification of detox juice preparation. Sixteen halophilic bacterial strains were isolated from the rhizospheric soil of *Arthrocnemum macrostachyum*. The isolates were screened for pectinase activity, and two strains, ASA21 and ASA29, exhibited the highest pectinase production in the presence of 2.5% NaCl, reaching 13.3 and 14.1 IU mL^−1^, respectively. The strains were identified as *Bacillus paralicheniformis* and *Paenibacillus* sp. by 16S rDNA sequencing and phylogenetic analysis. Growth kinetics and pectinase production studies revealed that both strains produced pectinase during the log phase, with ASA29 demonstrating higher growth and pectinase titers. The pectinase from ASA29 exhibited enhanced activity in the presence of 3% NaCl. The pectinases from both strains were applied for the clarification of detox juice prepared from beetroot, carrots, and apples. The use of 20 IU mL^−1^ pectinase from ASA29 for 2–3 h yielded > 96% juice with high total phenolic content and antioxidant activities. This study highlights the potential of salt-tolerant pectinolytic bacteria from the rhizosphere for biotechnological applications, particularly in the clarification of juices with high salt content.

## 1. Introduction

The rhizosphere is a soil region that has direct contact with the plant root system and hence receives various metabolites from plants and microorganisms. Therefore, it is a unique ecosystem where plants and microbes demonstrate a complex set of interactions [1]. This unique ecosystem harbors diversified microbial strains that utilize plant metabolites present in the root exudates [2]. Some of these plant-derived compounds can modulate the microbial system by acting as signal molecules [3]. The survival of a particular microbial species depends on the type of plant, its growth stage and soil conditions [4]; nonetheless, rhizospheric soil is rich in members of the phyla Proteobacteria, Actinobacteria, Bacteroidetes, and Firmicutes [5]. In addition, a high degree of spatial and temporal heterogeneity is also observed across root zones, rhizoplanes, and rhizosphere soil [6]. Lately, metagenomic studies affirmed the role of unidentified and uncultured microbial strains in the degradation of various substrates, including xenobiotics, plastics, and plant cell wall components [7]. In this context, bacterial species belonging to the genera *Bacillus, Anoxybacillus,* and *Streptomyces* have been reported for their ability to degrade plant biomass composed of lignin, cellulose, xylan and pectin [8].

Among rhizospheric bacteria, *Bacillus paralicheniformis*, a Gram-positive, spore-forming bacterium, has been reported to produce various extracellular enzymes [9] including cellulase, hemicellulase, and pectinase [10]. The cellulase from *B. paralicheniformis* acts synergistically with hemicellulases and hence can degrade plant biomass efficiently [11,12]. In the rhizosphere, this degradation of cellulosic and hemicellulosic contents renders the nutrients available to the plant, thereby enhancing plant growth [13]. However, pectinase production by this organism has been less frequently reported.

Another significant rhizospheric bacterial genus includes *Paenibacillus*, which has been reported for its wide-ranging metabolic capabilities [14]. The diversified activities exhibited by the members of this genus include nitrogen fixation, the production of bioactive compounds such as antibiotics, enzymes, and plant growth-promoting substances and the degradation of organic matter [15,16]. *Paenibacillus* species demonstrate their degradative potential by exhibiting various enzymes, including a complex cellulase system composed of endoglucanases, exoglucanases (cellobiohydrolases), and β-glucosidases [17]. It is believed that the rhizosphere lifestyle of this organism has bestowed it with the ability to degrade and assimilate complex plant materials [18]. Pectinolytic strains of *Paenibacillus* were reported previously for their application to improve the firmness of pineapple cubes [19], improve the clarification of grape, apple and orange juices [20] and extract pectin from apple pomace [21].

Pectin is an important component of plant biomass, particularly in softwoods and fruit peels. In addition to its various applications, the presence of high levels of structural constituents, such as pectin complexes in fruits and vegetable, impedes the extraction of their juices. Therefore, pectin removal is essential to improve extraction efficiency and to enhance clarification [22]. The enzymatic removal of pectin is popular and reportedly efficient because it maintains the high quality of the product [23,24]. Pectinase treatment results in a higher juice yield, the preservation of nutritional content, and enhanced production efficiency [25]. Pectinases cleave glycosidic bonds in polygalacturonic acid or related substrates and primarily release galacturonic acid and other compounds [26].

Pectinases from yeasts and molds hold the major share of the pectinase market, particularly for their applications in fruit juice clarification [27] where this enzyme degrades suspended particles of pectic compounds and improves juice clarity [28]. This pectinolytic treatment of juice imparts a high level of total soluble solids (TSSs) and sugars and enhances color [11]. This juice treatment process depends on various factors; however, enzyme loading remains a chief contributing factor [11]. The use of pectinases for a mix of fruit and vegetable juice has been described less frequently [25], particularly for vegetables containing high salt concentrations due to the salt sensitivity of the enzymes [23,25]. Considering their health benefits, fruit–vegetable mix juices are gaining popularity, owing to their unique ‘detox’ properties. As halophilic bacteria and salt-tolerant pectinases are not abundant [25], therefore, this study aimed to isolate salt-tolerant pectinolytic bacteria from the rhizospheric region of a salt marsh plant and to use pectinases from these bacteria for the clarification of a detox preparation comprising beetroot, carrot, and apple. Pectinase has not been utilized previously for the preparation of a mix or detox juice. It is imperative to consider the high salt concentration in beetroot that necessitates the use of salt-tolerant pectinase.

## 2. Methods

### 2.1. Reagents and Chemicals

All the chemicals and reagents were purchased from Sigma-Aldrich (St. Louis, MO, USA) and Daejung (Siheung-si, Republic of Korea). The chemicals were of analytical grade. Microbiological media were procured from Oxoid (Basingstoke, UK).

### 2.2. Isolation of Bacteria from Rhizospheric Soil

Halophilic bacterial strains were isolated from rhizospheric soil around the thick vegetation of Arthrocnemum macrostachyum near the coastal area of Karachi (map coordinates 27.096003, 65.831598). Soil samples were collected from the upper 15 cm layer around the root zone of healthy plants using sterile tools. The samples were stored at 4 °C and shipped to the laboratory within 24 h. A 10 g portion of each soil sample was suspended in 90 mL of sterile phosphate-buffered saline (PBS) and vortexed for 10 min to dislodge bacteria from soil particles. The suspension was serially diluted up to 10^−6^ in sterile PBS, and 100 µL aliquots from each dilution were spread onto nutrient agar plates and incubated at 30 °C for 24–48 h. Distinct bacterial colonies were selected based on their morphological characteristics for further purification and testing. The isolated bacterial strains were stored in (50%) glycerol stock at −20 °C.

### 2.3. Screening for Pectinase Activity

The isolated bacteria were screened for pectinase activity on pectin agar plates. The plates were prepared by adding 0.5% (*w*/*v*) citrus pectin to nutrient agar. The bacterial isolates were inoculated onto the plates and incubated at 30 °C for 48 h. After incubation, the plates were flooded with 1% (*w*/*v*) ruthenium red solution for 15 min. The formation of clear zones around the colonies indicated pectinase activity. The pectinolytic index (CI) was calculated by dividing the diameter of the clear zone by that of the bacterial colonies.

### 2.4. Molecular Identification Through 16S rDNA Sequencing

Two promising strains, ASA21 and ASA29, were identified by sequencing their 16S ribosomal rRNA genes. Bacterial genomic DNA was extracted using a commercial DNA extraction kit following the manufacturer’s instructions. The 16S rDNA region was amplified by PCR using universal primers 27F (5′-AGAGTTTGATCCTGGCTCAG-3′) and 1492R (5′-GGTTACCTTGTTACGACTT-3′). The PCR conditions were set as follows: initial denaturation at 95 °C for 5 min, followed by 35 cycles of 95 °C for 30 s, 55 °C for 30 s, and 72 °C for 90 s, with a final extension at 72 °C for 7 min. The amplified products were purified and sequenced. The obtained sequences were compared against the NCBI GenBank database using the BLASTn tool to identify their closest relatives.

### 2.5. Phylogenetic Analysis

The 16S rDNA sequences of the bacterial isolates were aligned with reference sequences from related species obtained from GenBank. Mega 11 software was used to construct the phylogenetic tree. The alignment of these sequences was performed using ClustalW and maximum likelihood was created with 1000 bootstrap replications. The Kimura-2-parameter was used as an evolutionary model.

### 2.6. Investigating Salt Tolerance

The salt tolerance of the bacterial isolates was assessed by growing them in nutrient broth supplemented with varying concentrations of NaCl. The isolates were incubated at 30 °C for 24 h, and growth was monitored by measuring the optical density at 600 nm (OD_600_) using a spectrophotometer. Salt tolerance was determined by comparing growth at different NaCl concentrations to the control (0% NaCl).

The salt tolerance of the pectinase enzyme produced by the selected strains was also evaluated. Pectinase preparations were incubated in buffer solutions containing different NaCl concentrations at 37 °C for 1 h. Residual pectinase activity was measured using the dinitrosalicylic acid (DNS) method [29].

### 2.7. Growth and Pectinase Production Kinetics

The growth kinetics of the selected bacterial strain were studied in a defined medium (Hassan et al., 2023 [26]) containing 1% (*w*/*v*) pectin as the sole carbon source. The bacterial culture was inoculated by maintaining 0.3 OD_600_ in 100 mL of medium and incubated at 30 °C with shaking at 150 rpm. Samples were taken at regular intervals, and growth was monitored by measuring the OD_600_. The specific growth rate (µ) was also calculated. The collected aliquots were centrifuged, and the supernatant was assayed for pectinase activity. A plot of OD600 or pectinase IU mL^−1^ against time was used to determine the growth phases, μ and volumetric productivity (IU L^−1^ h^−1^) of pectinase.

### 2.8. Juice Preparation

Beetroot, carrots, and apples were purchased from the local market. These materials were inspected for physical damage or apparent microbial degradation. Intact vegetables and fruits were packed in sealed containers until use. The juice was extracted following a previously reported method [30]. The dust and other impurities present in vegetables or fruit were removed by washing with tap water. The ingredients were cut into pieces, and the juice was extracted using a commercial blender (Panasonic, Hanoi, Vietnam) with 400 mL of purified (RO) water. The juice samples were stored in screw-capped bottles. To homogenize the juice, a domestic eggbeater (Panasonic) was used. Aliquots of well-mixed juice (20 mL) were collected every minute and transferred to screw-capped bottles. The juices were supplemented with 10 or 20 IU mL^−1^ of pectinase and kept at 30 °C for 2 or 3 h. The control samples were treated with a volume of distilled water equivalent to the enzyme loading. Afterwards, the samples were pasteurized at 90 °C for 5 min to halt pectinase activity and denature it. All juices were kept in a refrigerator until analysis.

### 2.9. Characterization of Detox Juice Formulation

#### 2.9.1. Juice Yield

Juice yield (%) was estimated using the following equation:

Yield (%) = m_2×C_/m_1×(100−w)_ × 100%

where m_1_ is the smash weight (g), m_2_ is the weight of vegetables and fruit (g), C represents the concentration of solvable compounds in the juice, % (*w*/*w*) and _w_ denotes the moistness of the initial content (%).

#### 2.9.2. Total Soluble Solids (°Bx)

An Antago RX-5000 digital refractometer (Tokyo, Japan) was used to estimate the total dissolved solids in the juice. The equipment was calibrated using distilled water at 0 °Bx, and the test solutions were analyzed to obtain degrees Brix (°Bx) representing dissolved solids (%).

#### 2.9.3. Estimation of Total Phenolic Content

The total phenolic content (TPC) was determined using the Folin–Ciocalteu method.

To extract total phenolics [30], 5 mL of the juice sample was mixed with 20 mL of 1.2 M HCl in 50% methanol. The mixture was incubated in the dark at 60 °C with shaking for 2 h. The supernatant was collected by centrifuging the contents at 3000 g for 15 min and stored at −20 °C.

The total phenolic content in the detox preparation was measured using the Folin–Ciocalteu (FC) assay. Briefly, the juice (100 µL) was mixed with 2 mL distilled water, 200 µL Folin–Ciocalteu reagent (Sigma-Aldrich), and 600 µL 20% sodium carbonate. The contents were blended for homogeneity, and the volume was made up to 5 mL using distilled water. The well-mixed solution was maintained at 50 °C for 30 min. The absorbance at a wavelength of 725 nm was noted using a UV–visible spectrophotometer. In the process of standard preparation, Gallic acid 0.01 to 0.05 mg mL^−1^ (Merck, Rahway, NJ, USA) prepared in 1.2 M hydrochloric acid methanolic solution was used to prepare control tubes. The phenolic content was expressed as milligrams of gallic acid equivalents (mgGAE) per 100 mL of juice.

#### 2.9.4. Estimation of Free Radical Scavenging Activity

The free radical scavenging activity of the juice sample was determined using two different methods, that is, DPPH and ABTS assays.

Briefly, DPPH solution was prepared (0.033%) and mixed with the juice in an equi-volume quantity. The mixture was placed at room temperature in the dark for 30 min. The absorbance at 517 nm was determined using a UV–visible spectrophotometer (Shimadzu 1780, Kyoto, Japan) with methanol as the blank. The IC_50_ value of each juice extract was determined. The percentage of free radical scavenging activity was calculated using the following formula:

Percent Scavenging activity = Absorbance of control − Absorbance of sample/Absorbance of control × 100

The antioxidant potential of the juice was also determined by ABTS reducing activity. The juice sample (100 µL) was mixed with 5 mL of 7 mM ABTS, and the mixture was kept at room temperature for 5–7 min. The absorbance of the mixture was measured at 734 nm against a blank. The IC_50_ value for each juice extract was also calculated.

### 2.10. Statistical Analysis

All experiments are performed in triplicate and the data are presented as the mean. The standard deviation of the data was calculated using Origin Lab 2024 software (Origin Lab (Northampton, MA, USA). To determine the effect of pectinase units and the time of treatment on juice parameters, two-way ANOVA was employed using SPSS version 21.

## 3. Results

The rhizosphere hosts metabolically diversified bacteria that find various biotechnological applications. The rhizosphere region around the halophytes is even differently enriched and found to be a source of salt-tolerant bacterial species. This study focused on obtaining salt-tolerant pectinolytic bacteria for prospects in industrial applications. 

### 3.1. Isolation and Screening of Halophilic Bacterial Strains for Pectinase Production

In all, 16 halophilic colonies were isolated from the rhizospheric region. The presence of distinct colonies in the presence of salt indicated the salt tolerance of the isolated species. The isolates were initially tested for pectinase production in the absence of salt. The strains that exhibited pectinase production in the plate assay were further screened for pectinase production in the presence of increasing salt concentrations. Four of the isolates (AA1, AA4, AA5 and AA11) did not exhibit any pectinolytic activity; therefore, they were not tested further. The 12 isolates were grown in the presence of varying salt concentrations in mineral medium containing pectin, and pectinase activity was assayed. 

Among all the isolates, isolates ASA21 and ASA29 exhibited the highest pectinase production in the presence of 2.5% NaCl, reaching 13.3 and 14.1 IU mL^−1^, respectively (Table 1). The rest of the isolates produced pectinase either in the presence of 1.5% NaCl or the titers remained low (<2 IU mL^−1^). Indeed, pectinase production was not detected in many isolates in the presence of salt concentrations of 2.5% or higher. Hence, isolates ASA21 and ASA29 were used for further experiments.

### 3.2. Identification of Promising Strains

**Figure 1:**
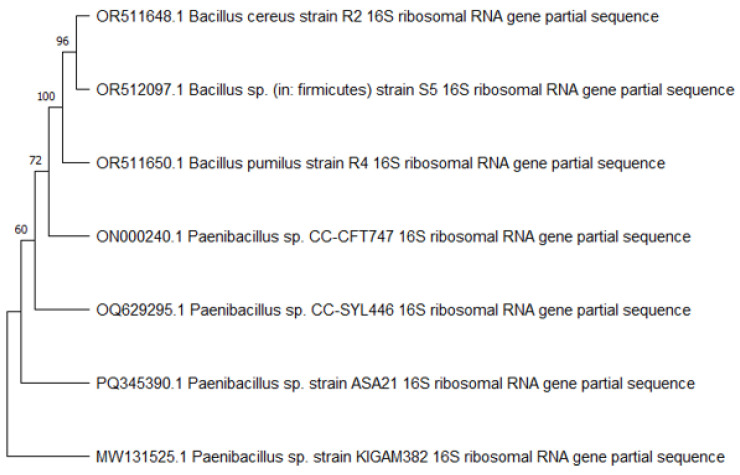
Phylogenetic analysis of the strain ASA21 (accession number PQ345390.1).

As strains ASA21 and ASA29 were promising pectinase-producing strains, they were identified through the amplification of 16S rDNA and phylogenetic analysis. The sequence data of strain ASA21 (Table S1, Supplementary File) was submitted with an accession number of PQ345390.1. Phylogenetic analysis revealed its clade with *Paenibacillus* species (Figure 1). Strain ASA21 was found to be closely associated with strains CC-SYL446 and CC-CFT747 of *Paenibacillus* sp., which were isolated from agricultural soil and yogurt, respectively.

**Figure 2:**
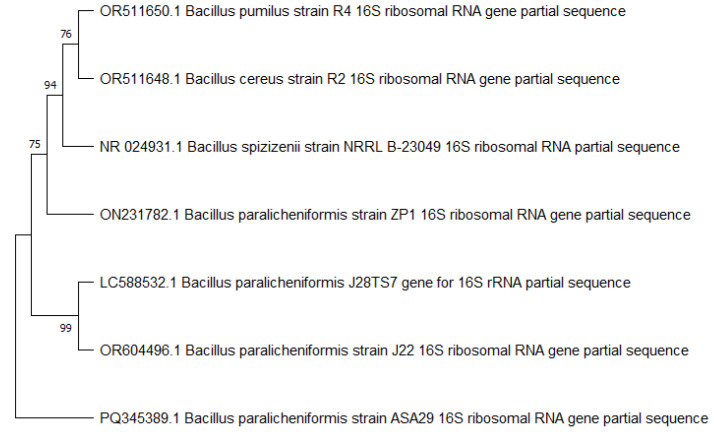
Phylogenetic analysis of the strain ASA29 (accession number PQ345389.1).

Strain ASA29 (accession number PQ345389.1) was identified as *B. paralicheniformis*. The nucleotide sequence of the 16S ribosomal RNA of this strain is given in Supplementary Table S2. Phylogenetic analysis revealed its close association with several strains of the same species including J28TS7, J22 and ZP1, which were isolated from Japanese honey, Pakistan honey and Serbia honey, respectively (Figure 2).

### 3.3. Growth and Pectinase Production Kinetics in Presence of Salt

**Figure 3:**
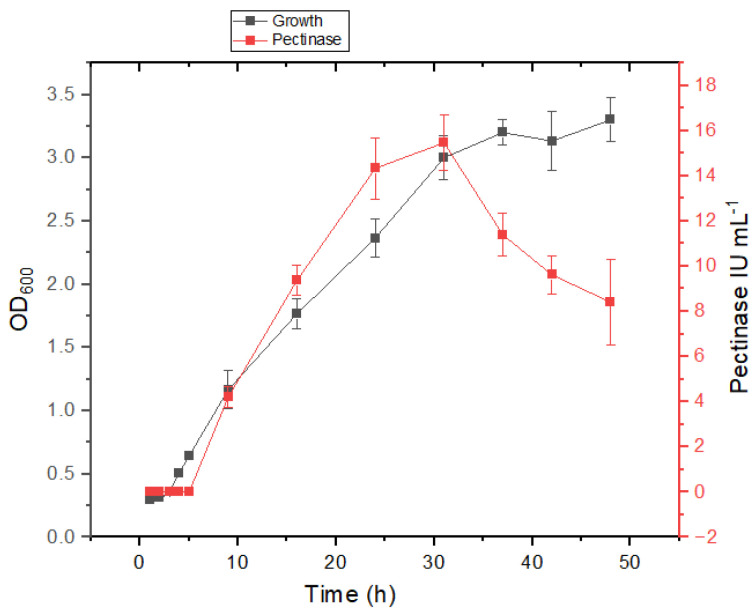
Growth and enzyme production kinetics of strain ASA21 in mineral salt medium supplemented with 1% pectin and 2.5% sodium chloride.

**Figure 4:**
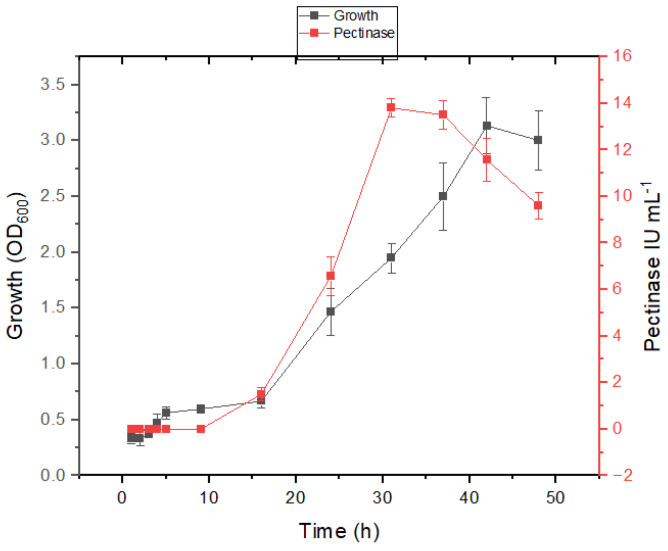
Growth and enzyme production kinetics of strain ASA21 in mineral salt medium supplemented with 1% pectin.

The isolates ASA21 and ASA29 demonstrated different behavior in salt-containing medium; however, a growth-linked production of pectinase was evident in both the isolates. The commencement of the log phase of the strain ASA21 was delayed for 3.3 h in salt-containing medium compared to 4 h (Figure 3) in minimal medium without salt (Figure 4). This indicated that the isolate was well adapted to grow in the presence of salt. In both media, pectinase production commenced in the early log phase and peaked in the late log phase, indicating the growth-associated expression of pectinase.

**Figure 5:**
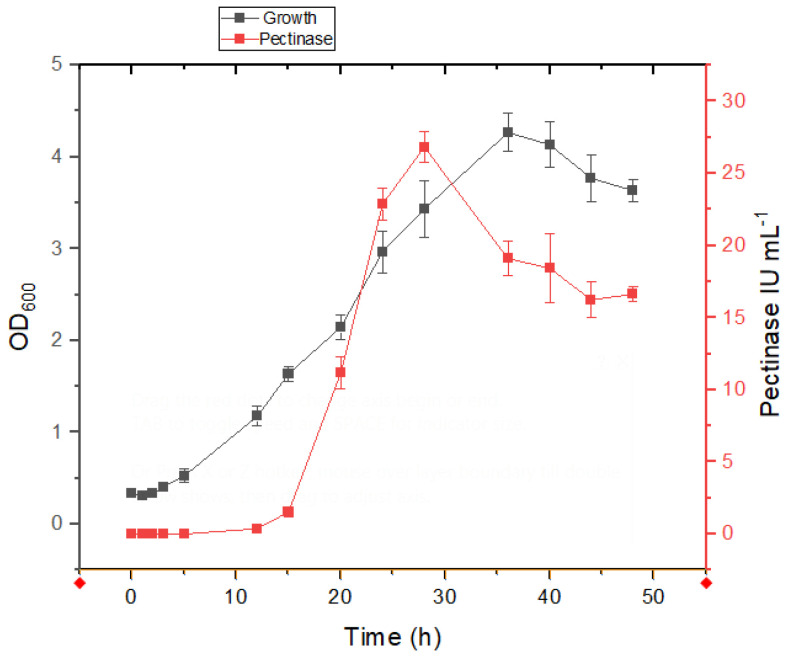
Growth and enzyme production kinetics of strain ASA29 in mineral salt medium supplemented with 1% pectin and 2.5% sodium chloride.

**Figure 6:**
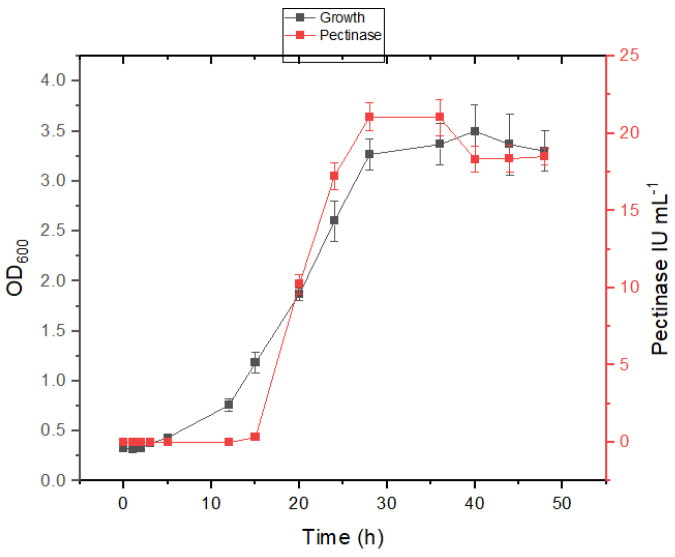
Growth and enzyme production kinetics of strain ASA29 in mineral salt medium supplemented with 1% pectin.

Strain ASA29 exhibited slightly different behavior, as pectinase production was delayed until the mid-log phase; however, it quickly reached its peak, producing more than 25 IU mL^−1^ in mineral salt medium containing 2.5% sodium chloride (Figure 5) and ~21 IU mL^−1^ in the absence of sodium chloride (Figure 6). Nonetheless, strain ASA29 demonstrated higher growth and pectinase titers than strain ASA21.

The specific growth rate was higher in salt-containing medium for both strains (Table 2). Nonetheless, strain ASA29 was a fast grower with a growth rate of 0.08 h^−1^. The volumetric productivity (Q_p_) of the two strains was also compared in the presence and absence of salt. Strain ASA29 demonstrated the highest productivity (925.9 IU L^−1^ h^−1^) in the presence of salt, affirming the halophilic nature of the strain (Table 2). The strain also manifested higher productivity (640 IU L^−1^ h^−1^) in the presence of salt.

### 3.4. Effect of Salt Concentration on Pectinase Activity Caused by Strains ASA21 and ASA29

**Figure 7:**
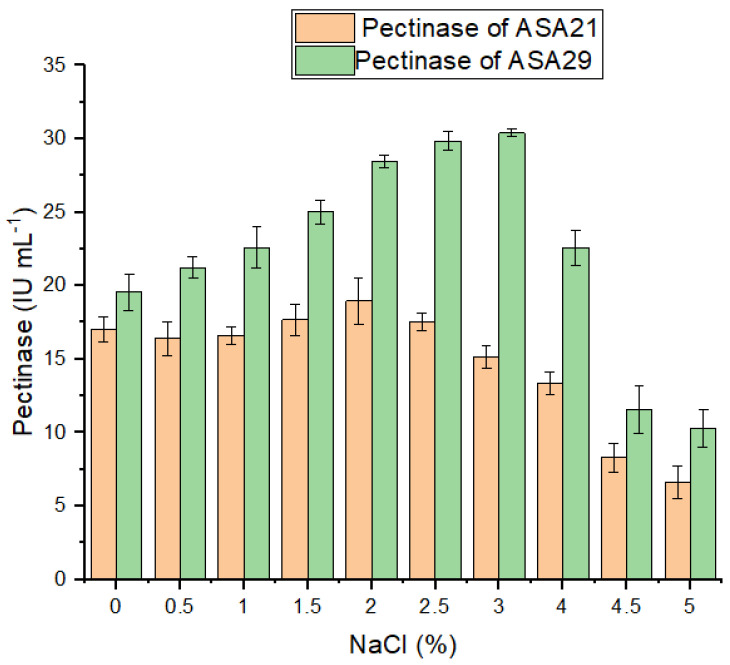
Effect of salt concentration on pectinase activity.

To investigate the impact of different concentrations of NaCl on the activity of the pectinase of strains ASA21 and ASA29, the crude enzyme preparations were dialyzed separately, and the IU mL^−1^ of pectinase was considered 100%. The varying salt concentrations were added separately to each preparation, and pectinase activity was determined. The pectinase activity of ASA21 was not enhanced by increasing the salt concentrations in the reaction mixture, although it tolerated up to 2.5–3% of salt (Figure 7). A further increase in salt concentration remarkably reduced the pectinase activity of ASA21.

The pectinase of strain ASA29, however, exhibited a different pattern as its activity increased with an increase in salt concentration up to 3%. The activity was enhanced 1.5 fold in the presence of 3% NaCl. However, a decrease of 35–40% in activity was observed when the salt concentration was further increased.

### 3.5. Clarification of Beetroot, Carrot and Apple Mix Juice

The application of 10 IU mL^−1^ pectinase of strain AA21 for 2 h yielded 43.8% yield (Table 3), which increased to more than 80% either by increasing the units of the enzyme to 20 or by increasing the duration to 3 h. The total phenolic content increased to a maximum of 44.8 mg/100 mL when 20 IU of pectinase was applied for 3 h. The antioxidant activities remained high when 20 units of pectinase were applied for 2 h and increased slightly when the juice clarification was extended for 3 h. The effect of pectinase units on strain ASA21 and the time of treatment was also investigated using two-way ANOVA (Table S3, Supplementary Material). The analysis showed that pectinase units and the time of treatment had a significant effect both individually and combined (Table S4, Supplementary Material). The juice attributes including yield, Brix value, and TPC were significantly affected by the pectinase units and the time of treatment (Table S5, Supplementary Material). Conversely, ABTS values remained insignificant when subjected to the combined effect of pectinase and the time of treatment.

Pectinase from strain AA29 performed better than AA21 when applied for juice clarification. In this case, the impact of the enzyme loading was evident as the use of 20 IU mL^−1^ yielded >96% of the juice either used for 2 or 3 h. This yield was concomitant with the high total phenolic content and antioxidant activity (Table 4). For this enzyme, the clarification reaction with 20 IU mL^−1^ for 2 h appeared to be better than the other combinations. 

The effect of dependent variables (pectinase from ASA29 and the time of treatment), individually and combined, was also investigated using two-way ANOVA (Table S6, Supplementary Material). Both variables had significant effect on the process of juice clarification (Table S7, Supplementary Material). All the juice attributes other than the antioxidant potential were significantly affected by the combined effect of pectinase and time (Table S8, Supplementary Material).

## 4. Discussion

The rhizosphere is a metabolically diversified region and has been proven to be a rich source of novel bacterial strains. The rhizosphere of halophytes, particularly salt marshes, has been reported to inhabit microbial strains capable of producing industrially important enzymes. This study reported the isolation of salt-tolerant bacterial species from the rhizosphere region of a salt marsh plant. Salt-tolerant strains have received more attention for their prospects in biotechnological applications and saline agriculture. Tirry et al. [31] isolated four salt-tolerant plant growth-promoting strains and reported a positive impact of these strains on the salt tolerance and cultivation of the plant *Medicago sativa*, indicating the potential use of salt-tolerant species in promoting the growth of glycophytes in brackish land. In this study, promising salt-tolerant strains with the ability to produce pectinase were identified as *B. paralicheniformis* and *Paenibacillus* sp. *B. paralicheniformis*, a Gram-positive facultative anaerobic motile bacterium, has been differentiated from other closely related *Bacillus* species such as *B. licheniformis* and *B. sonorensis* on the basis of genetic clusters and has been described for its versatile biotechnological applications [32,33]. Rhizosphere has been reported as its habitat; for instance, *B. paralicheniformis* 2R5 was isolated from the canola rhizosphere and was reported to have plant growth-promoting abilities [34]. Through genomic analysis, Ngom et al. [35] reported the presence of genes in *B. paralicheniformis* responsible for the degradation of various hemicellulosic moieties, including xylan and pectin. Rahman et al. [36] used polygalacturonase from this organism for the degumming of ramie fiber. In this study, the strain ASA29 of *B. paralicheniformis* demonstrated salt tolerance with the ability to produce pectinase in salt-containing medium. 

*Paenibacillus* species have also been reported in the rhizospheric region. Da Silva et al. [37] adopted the denaturing gradient gel electrophoresis (DGGE) approach to investigate the diversity of *Paenibacillus* sp. in maize rhizosphere and discerned the presence of different *Paenibacillus* species in this region. Various authors have reported nitrogen fixation and other plant growth-promoting properties of *Paenibacillus* species [38,39]. Pectinase production from this organism has also been described [40,41]. In this study, the rhizospheric strain, ASA21 of *Paenibacillus* sp., showed its ability to produce pectinase in salt-containing medium.

This study reported the production of pectinase in the log phase with a Q_p_ of 925 and 640 IU L^−1^ h^−1^ pectinase in the presence of 2.5% salt. Previously, the maximum cell density of *B. paralicheniformis* was reported in 28 h, with the highest productivity (Q_p_) of alkaline phosphatase in 181.78 U L^−1^ h^−1^ [42]; a change in volumetric productivity was observed with a change in pH and nutrients.

Our study demonstrated that strains ASA21 and ASA29 remain for 2–5 h in the lag phase of their growth in the presence or absence of salt. In another study, a lag phase of 10 h for *B. paralicheniformis* was reported in the presence or absence of selenite when the OD_600_ of the culture was traced and the strain followed a ~10 h log phase, as indicated by a rapid increase in the absorbance [43]; however, this growth was noticeably slowed down when the medium was supplemented with more than 1% NaCl. Conversely, the strains studied here demonstrated considerable growth, even in presence of 2.5% salt, indicating the salt-tolerant nature of these bacterial strains. A high salt tolerance in *B. licheniformis* strain was reported by Zhang et al. [44] for an esterase-producing strain. 

The variation in the duration of the growth phases has also been reported previously by Li et al. [45], when the growth kinetics of a gamma-glutamyl peptide-producing strain of *B. paralicheniformis* was found to vary slightly with a change in the growth medium. The organism remained in the log phase for a day, followed by a 3–4-day-long stationary phase. This difference can be attributed to the presence of media components, particularly trace elements. In comparison to this reported strain, strain ASA29 appeared as a fast-growing organism, attaining its peak growth in less than 48 h, even in the presence of salt.

Gummadi et al. [46] isolated a salt-tolerant yeast, *Debaromyces nepalensis* NCYC3413, from a rotten apple; the pectinase from this yeast exhibited optimal activity in presence of 2M NaCl. The salt tolerance of the yeast strain showed its ability to sustain a toxic effect, possibly due to a modified Na/K channel. The salt tolerance exhibited by the pectinase of ASA21 and ASA29 may be attributed to their tertiary structure; however, this conclusion needs further investigation. Salt-tolerant enzymes find various biotechnological applications; for instance, De Paula et al. [47] reported laccase from salt-tolerant fungal strains for the degradation of textile effluent. Therefore, pectinases from ASA21 and ASA29 warrant further investigations to widen their prospects in industries.

An esterase from a salt-tolerant strain exhibited optimal activity in the presence of 3.5 M NaCl, and the authors linked the presence of more alpha-helices and fewer beta-sheets to this novel salt-tolerance [48]. In this study, pectinase from ASA29 was activated in the presence of salt and showed maximum activity in the presence of 2.5% salt. However, the mechanism involved in salt tolerance needs to be investigated at the protein level. Nonetheless, pectinases that can withstand extreme conditions, such as high salt concentrations, are of particular interest for various industrial applications, including the production of prebiotics, the retting of plant fibers and tea and coffee fermentation [48,49].

In this study, 20 IU mL^−1^ of pectinase from AA29 yielded >96% of the mixed juice when the process was carried out for 2–3 h. This yield was better than that reported by Mahto et al. [23] for a 79.25, 72.76 and 61.24% clarification of sweet lime, pineapple and lime juices, respectively, using pectinase from a mutant strain of *B. subtilis.* However, in this study, we also observed an improvement in the antioxidant potential of the juice, with a significant quantity of total phenolics. Bioactive compounds such as total phenolics present in juices can boost the immune system and have been reported to have cardio-protecting effects. Previously, Vasconcellos et al. [50] reported 3.7 mg g^−1^ of total phenolics in beetroot juice [51]. Total phenolics in beetroot can be categorized as phenolic acids and flavonoids [52]. However, the amount of these bioactive compounds varies greatly, depending on the variety of fruits or vegetables, ripening stage, and extraction process [52,53]. Therefore, a mix of juices is desirable to obtain a blend of different bioactive compounds. Indeed, it has been reported that juices extracted from a mix of fruits exhibit higher antioxidant potential than juices from a single source [54]. This study also reported the antioxidant potential of mixed juice extracted using pectinase preparations. However, a complete chemical analysis is required to identify the compounds responsible for these activities. 

## 5. Conclusions

The rhizosphere of the salt marsh plant *Arthrocnemum macrostachyum* exhibited a promising source of salt-tolerant pectinolytic bacteria. *Paenibacillus* sp. ASA21 *Bacillus licheniformis* ASA29 are fast growing strains that can resist 2.5% salt and produce salt-tolerant pectinase. The tolerance of pectinase to salt supports its application in the clarification of mixed juice preparations. The pectinases from these two strains improved the extraction of a mixed or detox juice composed of beetroot, apple and carrot. The pectinase-mediated treatment of the juice improved the yield from 42 to 90%, total phenol contents from 22 to 52 mgGAE/100 mL, and antioxidant potential. The analysis of the juice for the identification of these bioactive compounds can provide insights for the future applications of these juices. 

## Figures and Tables

**Table 1:** Pectinase production in the presence of varying amounts of NaCl.

Isolate	Pectinase Production (IU mL^−1^) in Mineral Salt Medium Containing NaCl (%)					
	0	1.0	1.5	2.0	2.5	3.0
ASA3	3.7 (±0.21)	4.1 (±0.18)	1.8 (±0.11)	0.6 (±0.47)	0 (±0)	0 (±0)
ASA8	7.8 (±0.42)	6.5 (±0.09)	7.1 (±0.68)	2.8 (±0.54)	1.2 (±0.21)	0 (±0)
ASA9	1.2 (±0.27)	0.7 (±0.06)	0.5 (±0.01)	0 (±0)	0 (±0)	0 (±0)
ASA14	6.1 (±0.31)	5.8 (±0.47)	2.1 (±0.08)	0.6 (±0.11)	0 (±0)	0 (±0)
ASA19	2.1 (±0.17)	4.1 (±0.19)	3.9 (±0.26)	1.2 (±0.19)	0.9 (±0.10)	0.5 (±0.03)
ASA20	5.3 (±0.22)	3.1 (±0.25)	2.5 (±0.38)	0.6 (±0.17)	0 (±0)	0 (±0)
ASA21	11.8 (±0.86)	12.1 (±1.01)	12.6 (±0.87)	11.9 (±0.90)	13.3 (±0.71)	8.2 (±0.76)
ASA22	9.1 (±0.98)	8.5 (±0.77)	8.2 (±1.1)	9.2 (±0.76)	6.3 (±0.78)	2.4 (±0.55)
ASA24	6.1 (±0.91)	5.1 (±0.28)	3.2 (±0.41)	3.1 (±0.78)	2.7 (±0.01)	1.1 (±0.12)
ASA26	1.1 (±0.12)	1.8 (±0.17)	2.1 (±0.29)	2.4 (±0.34)	0.6 (±0.47)	0 (±0)
ASA29	13.2 (±0.08)	13.9 (±0.92)	13.9 (±0.71)	13.5 (±0.77)	14.1 (±0.85)	10.2 (±0.11)
ASA31	4.5 (±0.59)	4.1 (±0.72)	4.1 (±0.46)	3.5 (±0.13)	3.1 (±0.31)	1.7 (±0.19)

**Table 2:** Specific growth rate (μ) and volumetric productivity (Q_p_) of pectinase by the strains ASA21 and ASA29 in mineral salt medium containing 2.5% NaCl and without NaCl.

Strain	μ in Mineral Salt Medium (h^−1^)		Q_p_ (IU L^−1^ h^−1^) in the Mineral Salt Medium	
	Without NaCl	With 2.5% NaCl	Without NaCl	With 2.5% NaCl
ASA21	0.069 (±0.001)	0.081 (±0.001)	560 (±35)	640 (±51)
ASA29	0.83 (±0.002)	0.088 (±0.001)	625 (±45)	925.9 (±32)

**Table 3:** The effects of pectinase units from strain ASA21, the time of processing on the juice yield, total solids, total phenolic content (TPC), and antioxidant potential (presented as ABTS and DPPH). The control sample was processed without pectinase (0 units).

Pectinase Units	Time Duration (min)	Juice Yield (%)	Total Soluble Solids (°Bx)	TPC (mgGAE/100mL)	ABTS (IC_50_)	DPPH (IC_50_)
10	120	43.8 (±1.5)	12 (±1.01)	31.6 (±2.7)	40 (±3.1)	50 (±2.8)
10	180	81.4 (±7.1)	18 (±1.0)	42.3 (±2.7)	60 (±3.1)	55 (±4.1)
20	120	82.3 (±7.3)	20 (±1.9)	43.9 (±1.9)	70 (±3.5)	60 (±2.8)
20	180	81.1 (±6.0)	19 (±1.7)	44.8 (±3.7)	80 (±5.0)	60 (±3.5)
0	120	42.5 (±2.7)	8 (±0.9)	21.3 (±1.1)	35 (±1.8)	38 (±1.4)
0	180	43.8 (±3.5)	9 (±0.8)	22.7 (±1.8)	37 (±2.6)	38 (±1.9)

**Table 4:** The effects of pectinase units from strain ASA29, the time of processing on the juice yield, total solids, total phenolic content (TPC), and antioxidant potential (presented as ABTS and DPPH). The control sample was processed without pectinase (0 units).

Pectinase Units	Time Duration (min)	Juice Yield (%)	Total Soluble Solids (°Bx)	TPC (mgGAE/100mL)	ABTS (IC_50_)	DPPH (IC_50_)
10	120	65.7 (±4.8)	15 (±0.6)	45.7 (±2.2)	70 (±4.8)	70 (±3.4)
10	180	81.3 (±7.2)	24 (±3.5)	52.9 (±5.8)	80 (±7.4)	75 (±4.9)
20	120	96.4 (±6.1)	26 (±1.9)	54.2 (±5.6)	100 (±0.5)	100 (±0)
20	180	97.8 (±3.1)	27 (±0.8)	52.9 (±7.1)	100 (±0)	90 (±0.2)
0	120	42.5 (±2.7)	8 (±0.9)	21.3 (±1.1)	35 (±1.8)	38 (±1.4)
0	180	43.8 (±3.5)	9 (±0.8)	22.7 (±1.8)	37 (±2.6)	38 (±1.9)

## Data Availability

The raw data supporting the conclusions of this article will be made available by the authors on request.

## Supplementary Materials

The following supporting information can be downloaded at https://www.mdpi.com/article/10.3390/microorganisms12112162/s1. Table S1. Sequence of 16S rDNA of strain ASA21. Table S2. Sequence of 16S rDNA of strain ASA29. Table S3. Data analyzed to investigate effect of pectinase units of strain ASA21 and time duration of juice parameters. Table S4. Multivariate tests performed to investigate the effect of individual and combined factors for the treatment of juice by the pectinase of strain ASA21. Table S5. Effect of pectinase from ASA21 and treatment time on juice yield, Brix values, total phenol content (TPC), antioxidant potential (as ABTS and DPPH values). Table S6. Data analyzed to investigate effect of pectinase units of strain ASA29 and time duration of juice parameters. Table S7. Multivariate tests performed to investigate the effect of individual and combined factors for the treatment of juice by the pectinase of strain ASA29. Table S8. Effect of pectinase from ASA29 and treatment time on juice yield, Brix values, total phenol content (TPC), antioxidant potential (as ABTS and DPPH values).
