# Supplementary material for: Halophilic Pectinase-Producing Bacteria from Arthrocnemum macrostachyum Rhizosphere: Potential for Fruit–Vegetable Juice Processing
Source: Microorganisms. 2024 Oct 26;12(11):2162. doi: 10.3390/microorganisms12112162 (PMC11596074; doi:10.3390/microorganisms12112162)
Supplement: Supplementary file 1 [file microorganisms-12-02162-s001.zip › microorganisms-3276542-supplementary.pdf]

Supplementary file

Table S1. 16S rDNA sequence of the strain ASA21

CTGATGTAGCTTGCTACTGATGATGCTCAGCGGCGGACGGGTGAGTAACACGTA<br>GGCAACCTGCCTGCAAGACCGGGATAACCCACGGAAACGTGAGCTAATACCGG<br>ATAGATGGTCTCTTCGCATGGAGGGATCAGGAAAGACGGAGCAATCTGTCACTT<br>GCGGATGGGCCTGCGGCGCATTAGCTAGTAGGTGAGGTAACGGCTCACCTAGGC<br>GACGATGCGTAGCCGACCTGAGAGGGTGAACGGCCACACTGGGACTGAGACAC<br>GGCCCAGACTCCTACGGGAGGCAGCAGTAGGGAATCTTCGCAATGGACGCAAG<br>TCTGACCGAGCAACGCCGCGTGAGTGAAGAAGGGTTTCGGCTCGTAAAACTCT<br>GTTGCCAGGGAAGAACGCCGGTGAGAGTAACTGCTCACCGGGTGACGGTACCT<br>GAGAAGAAAGCCCCGGCTAACTACGTGCCAGCAGCCGCGGTAATACGTAGGGG<br>GCAAGCGTTGTCCGGAATTATTGGGCGTAAAGCGCGCGCAGGCGGTCGTTTAAG<br>TCTGGTGTTTAAACCCAAGGCTCAACCTTGGGTCGCACTAGAAACTGGGCGGCT<br>GGAGTGCAGGAGAGGAAAGTGGAATTCCACGTGTAGCGGTGAAATGCGTAGAG<br>ATGTGGAGGAACACCAGTGGCGAAGGCGACTTTCTGGCCTGTAACTGACGCTG<br>AGGCGCGAAAGCGTGGGGAGCAAACAGGATTAGATACCCTGGTAGTCCACGCC<br>GTAAACGATGAGTGCTAGGTGTTAGGGGTTTCGATACCCTTGGTGCCGAAGTTA<br>ACACAGTAAGCACTCCGCCTGGGGAGTACGCTCGCAAGAGTGAAACTCAAAGG<br>AATTGACGGGGACCCGCACAAGCAGTGGAGTATGTGGTTTAATTCGAAGCAATG<br>CGAAGAACCTTACCAGGTCTTGACATCTGGGTGAAACATGCAGAGATGTATGCC<br>TCCTTCGGGACACCCAAGACAGGTGGTGCATGGTTGTCGTCAGCTCGTGTCGTG<br>AGATGTTGGGTTAAGTCCCGCAACGAGCGCAACCCTTGATCTTAGTTGCCAGCA<br>TTGAGTTGGGCACTCTAGGATGACTGCCGGTGACAAACCGGAGGAAGGTGGGG<br>ATGACGTCAAATCATCATGCCCCTTATGACCGGGCTACACACGTACTACAATGGC<br>CGGTACAACGGGAAGCGAAGTGGCGACACGGAGCGAATCCTTAGAAGCCGGTC<br>TCAGTTCGGATTGCAGGCTGCAACTCGCCTGCATGAAGTCGGAATTGCTAGTAA<br>TCGCGGATCAGCATGCCGCGGTGAATACGTTCCCGGGTCTTGTACACACCGCCC<br>GTCACACCACGAGAGT

Table S2. 16S rDNA sequence of the strain ASA29

AGGCAACGATGCGTAGCCGACCTGAGAGGGTGATCGGCCACACTGGGACTGAG<br>ACACGGCCCAGACTCCTACGGGAGGCAGCAGTAGGGAATCTTCCGCAATGGAC<br>GAAAGTCTGACGGAGCAACGCCGCGTGAGTGATGAAGGTTTTCGGATCGTAAA<br>ACTCTGTTGTTAGGGAAGAACAAGTACCGTTCGAATAGGGCGGTACCTTGACGG<br>TACCTAACCAGAAAGCCACGGCTAACTACGTGCCAGCAGCCGCGGTAATACGTA<br>GGTGGCAAGCGTTGTCCGGAATTATTGGGCGTAAAGCGCGCGCAGGCGGTTTCT<br>TAAGTCTGATGTGAAAGCCCCCGGCTCAACCGGGGAGGGTCATTGGAAACTGG<br>GGAACTTGAGTGCAGAAGAGGAGAGTGGAATTCCACGTGTAGCGGTGAAATGC<br>GTAGAGATGTGGAGGAACACCAGTGGCGAAGGCGACTCTCTGGTCTGTAACTG<br>ACGCTGAGGCGCGAAAGCGTGGGGAGCGAACAGGATTAGATACCCTGGTAGTC<br>CACGCCGTAAACGATGAGTGCTAAGTGTTAGAGGGTTTCCGCCCTTTAGTGCTG<br>CAGCAAACGCATTAAGCACTCCGCCTGGGGAGTACGGTCGCAAGACTGAAACT<br>CGAAGGAATTGACGGGGGCCCGCACAAGCGGTGGAGCATGTGGTTTAATTCGA<br>AGCAACGCGAAGAACCTTACCAGGTCTTGACATCCTCTGACAACCCTAGAGATA<br>GGGCTTCCCCTTCGGGGGCAGAGTGACAGGTGGTGCATGGTTGTCGTCAGCTC<br>GTGTCGTGAGATGTTGGGTTAAGTCCCGCAACGAGCGCAACCCTTGATCTTAGT<br>TGCCAGCATTCAGTTGGGCACTCTAAGGTGACTGCCGGTGACAAACCGGAGGA

AGGTGGGGATGACGTCAAATCATCATGCCCCTTATGACCTGGGCTACACACGTG
CTACAATGGGCAGAACAAAGGGCAGCGAAGCCGCGAGGCTAAGCCAATCCCAC
AAATCTGTTCTCAGTTCGGATCGCAGTCTGCAACTCGACTGCGTGAAGCTGGAA
TCGCTAGTAATCGCGGATCAGCATGCCGCGGTGAATACGTTCCCGGGCCTTGTAC
ACACCGCCCGTCACACCACGAGAGTTTGTAACACCCGAAGTCGGTGAGGTAAC
CTTTTGGAGCCAGCCGCCGAAGGTGGGACAGATGATTGGGGTGAAGTCGTAAC
AAGGTAGCCG

Table S3. Data analyzed to investigate effect of pectinase units of strain ASA21 and time duration of juice parameters.

Between-Subjects Factors

		N
Pectinase units (Label: 1=0, 2=10, 3=20)	.00	6
	10.00	6
	20.00	6
Time duration (Label: 1=120, 2=180)	120.00	9
	180.00	9

Table S4. Multivariate tests performed to investigate the effect of individual and combined factors for the treatment of juice by the pectinase of the strain ASA21

Multivariate Tests[a]

Effect		Value	F	Hypothesis df	Error df	Sig.
Intercept	Pillai's Trace	1.000	8806.843[b]	5.000	8.000	.000
	Wilks' Lambda	.000	8806.843[b]	5.000	8.000	.000
	Hotelling's Trace	5504.277	8806.843[b]	5.000	8.000	.000
	Roy's Largest Root	5504.277	8806.843[b]	5.000	8.000	.000
Pectinase	Pillai's Trace	1.678	9.388	10.000	18.000	.000
	Wilks' Lambda	.001	52.339[b]	10.000	16.000	.000
	Hotelling's Trace	363.700	254.590	10.000	14.000	.000
	Roy's Largest Root	361.566	650.818[c]	5.000	9.000	.000
Time	Pillai's Trace	.969	49.798[b]	5.000	8.000	.000
	Wilks' Lambda	.031	49.798[b]	5.000	8.000	.000
	Hotelling's Trace	31.124	49.798[b]	5.000	8.000	.000
	Roy's Largest Root	31.124	49.798[b]	5.000	8.000	.000
Pectinase * Time	Pillai's Trace	1.394	4.141	10.000	18.000	.004
	Wilks' Lambda	.012	12.830[b]	10.000	16.000	.000
	Hotelling's Trace	47.285	33.100	10.000	14.000	.000
	Roy's Largest Root	46.576	83.836[c]	5.000	9.000	.000

a. Design: Intercept + Pectinase + Time + Pectinase * Time

b. Exact statistic

c. The statistic is an upper bound on F that yields a lower bound on the significance level.

Table S5. Effect of pectinase from ASA21 and treatment time on juice yield, Brix values, Total Phenol content (TPC), Antioxidant potential (as ABTS and DPPH values).

Tests of Between-Subjects Effects

Source	Dependent Variable	Type III Sum of Squares	df	Mean Square	F	Sig.
Corrected Model	Yield (%)	6299.871[a]	5	1259.974	331.863	.000
	Brix (Bx)	396.338[b]	5	79.268	95.121	.000
	TPC (mgGAE/100mL)	1718.378[c]	5	343.676	231.865	.000
	ABTS (IC50)	9513.378[d]	5	1902.676	13.022	.000
	DPPH (IC50)	1648.436[e]	5	329.687	66.821	.000
Intercept	Yield (%)	67466.889	1	67466.889	17770.032	.000
	Brix (Bx)	3738.242	1	3738.242	4485.891	.000
	TPC (mgGAE/100mL)	21231.736	1	21231.736	14324.259	.000
	ABTS (IC50)	75608.642	1	75608.642	517.472	.000
	DPPH (IC50)	46015.667	1	46015.667	9326.450	.000
Pectinase	Yield (%)	4536.081	2	2268.041	597.377	.000
	Brix (Bx)	346.484	2	173.242	207.891	.000
	TPC (mgGAE/100mL)	1534.314	2	767.157	517.572	.000
	ABTS (IC50)	9213.634	2	4606.817	31.529	.000
	DPPH (IC50)	1615.334	2	807.667	163.698	.000
Time	Yield (%)	664.909	1	664.909	175.130	.000
	Brix (Bx)	6.969	1	6.969	8.363	.014
	TPC (mgGAE/100mL)	86.242	1	86.242	58.184	.000
	ABTS (IC50)	184.320	1	184.320	1.262	.283
	DPPH (IC50)	14.401	1	14.401	2.919	.113
Pectinase * Time	Yield (%)	1098.881	2	549.441	144.717	.000
	Brix (Bx)	42.884	2	21.442	25.731	.000
	TPC (mgGAE/100mL)	97.821	2	48.911	32.998	.000
	ABTS (IC50)	115.423	2	57.712	.395	.682
	DPPH (IC50)	18.701	2	9.351	1.895	.193
Error	Yield (%)	45.560	12	3.797		
	Brix (Bx)	10.000	12	.833		
	TPC (mgGAE/100mL)	17.787	12	1.482		
	ABTS (IC50)	1753.340	12	146.112		
	DPPH (IC50)	59.207	12	4.934		
Total	Yield (%)	73812.320	18			
	Brix (Bx)	4144.580	18			
	TPC (mgGAE/100mL)	22967.900	18			
	ABTS (IC50)	86875.360	18			

	DPPH (IC50)	47723.310	18			
Corrected Total	Yield (%)	6345.431	17			
	Brix (Bx)	406.338	17			
	TPC (mgGAE/100mL)	1736.164	17			
	ABTS (IC50)	11266.718	17			
	DPPH (IC50)	1707.643	17			

a. R Squared = .993 (Adjusted R Squared = .990)

b. R Squared = .975 (Adjusted R Squared = .965)

c. R Squared = .990 (Adjusted R Squared = .985)

d. R Squared = .844 (Adjusted R Squared = .780)

e. R Squared = .965 (Adjusted R Squared = .951)

Table S6. Data analyzed to investigate effect of pectinase units of strain ASA29 and time duration of juice parameters.

Between-Subjects Factors

		N
Pectinase units (Label: 1=0, 2=10, 3=20)	.00	6
	10.00	6
	20.00	6
Time duration (Label: 1=120, 2=180)	120.00	9
	180.00	9

Table S7. Multivariate tests performed to investigate the effect of individual and combined factors for the treatment of juice by the pectinase of the strain ASA29

Multivariate Tests[a]

Effect		Value	F	Hypothesis df	Error df	Sig.
Intercept	Pillai's Trace	1.000	41945.961[b]	5.000	8.000	.000
	Wilks' Lambda	.000	41945.961[b]	5.000	8.000	.000
	Hotelling's Trace	26216.226	41945.961[b]	5.000	8.000	.000
	Roy's Largest Root	26216.226	41945.961[b]	5.000	8.000	.000
Pectinase	Pillai's Trace	1.799	16.094	10.000	18.000	.000
	Wilks' Lambda	.000	194.617[b]	10.000	16.000	.000
	Hotelling's Trace	3023.748	2116.624	10.000	14.000	.000
	Roy's Largest Root	3019.770	5435.585[c]	5.000	9.000	.000
Time	Pillai's Trace	.907	15.570[b]	5.000	8.000	.001
	Wilks' Lambda	.093	15.570[b]	5.000	8.000	.001

	Hotelling's Trace	9.731	15.570[b]	5.000	8.000	.001
	Roy's Largest Root	9.731	15.570[b]	5.000	8.000	.001
Pectinase * Time	Pillai's Trace	1.668	9.045	10.000	18.000	.000
	Wilks' Lambda	.007	17.236[b]	10.000	16.000	.000
	Hotelling's Trace	44.007	30.805	10.000	14.000	.000
	Roy's Largest Root	41.766	75.179[c]	5.000	9.000	.000

a. Design: Intercept + Pectinase + Time + Pectinase * Time

b. Exact statistic

c. The statistic is an upper bound on F that yields a lower bound on the significance level.

Table S8. Effect of pectinase from ASA29 and treatment time on juice yield, Brix values, Total Phenol content (TPC), Antioxidant potential (as ABTS and DPPH values).

Tests of Between-Subjects Effects

Source	Dependent Variable	Type III Sum of Squares	df	Mean Square	F	Sig.
Corrected Model	Yield (%)	9527.316[a]	5	1905.463	148.259	.000
	Brix (Bx)	1092.498[b]	5	218.500	145.075	.000
	TPC (mgGAE/100mL)	3470.116[c]	5	694.023	51.004	.000
	ABTS (IC50)	12515.600[d]	5	2503.120	900.943	.000
	DPPH (IC50)	10123.727[e]	5	2024.745	582.474	.000
Intercept	Yield (%)	89619.667	1	89619.667	6973.087	.000
	Brix (Bx)	5911.469	1	5911.469	3924.989	.000
	TPC (mgGAE/100mL)	30644.627	1	30644.627	2252.085	.000
	ABTS (IC50)	89126.420	1	89126.420	32079.095	.000
	DPPH (IC50)	84624.980	1	84624.980	24344.728	.000
Pectinase	Yield (%)	9078.564	2	4539.282	353.190	.000
	Brix (Bx)	950.724	2	475.362	315.622	.000
	TPC (mgGAE/100mL)	3383.574	2	1691.787	124.330	.000
	ABTS (IC50)	12354.190	2	6177.095	2223.310	.000
	DPPH (IC50)	9950.890	2	4975.445	1431.325	.000
Time	Yield (%)	215.627	1	215.627	16.777	.001
	Brix (Bx)	68.836	1	68.836	45.704	.000
	TPC (mgGAE/100mL)	10.427	1	10.427	.766	.399
	ABTS (IC50)	78.542	1	78.542	28.270	.000
	DPPH (IC50)	14.222	1	14.222	4.091	.066
Pectinase * Time	Yield (%)	233.124	2	116.562	9.069	.004
	Brix (Bx)	72.938	2	36.469	24.214	.000
	TPC (mgGAE/100mL)	76.114	2	38.057	2.797	.101
	ABTS (IC50)	82.868	2	41.434	14.913	.001
	DPPH (IC50)	158.614	2	79.307	22.815	.000

	Yield (%)	154.227	12	12.852		
	Brix (Bx)	18.073	12	1.506		
Error	TPC (mgGAE/100mL)	163.287	12	13.607		
	ABTS (IC50)	33.340	12	2.778		
	DPPH (IC50)	41.713	12	3.476		
	Yield (%)	99301.210	18			
	Brix (Bx)	7022.040	18			
Total	TPC (mgGAE/100mL)	34278.030	18			
	ABTS (IC50)	101675.360	18			
	DPPH (IC50)	94790.420	18			
	Yield (%)	9681.543	17			
	Brix (Bx)	1110.571	17			
Corrected Total	TPC (mgGAE/100mL)	3633.403	17			
	ABTS (IC50)	12548.940	17			
	DPPH (IC50)	10165.440	17			

a. R Squared = .984 (Adjusted R Squared = .977)

b. R Squared = .984 (Adjusted R Squared = .977)

c. R Squared = .955 (Adjusted R Squared = .936)

d. R Squared = .997 (Adjusted R Squared = .996)

e. R Squared = .996 (Adjusted R Squared = .994)
